# The structure of the RbBP5 β-propeller domain reveals a surface with potential nucleic acid binding sites

**DOI:** 10.1093/nar/gky199

**Published:** 2018-03-21

**Authors:** Anshumali Mittal, Fruzsina Hobor, Ying Zhang, Stephen R Martin, Steven J Gamblin, Andres Ramos, Jon R Wilson

**Affiliations:** 1 The Francis Crick Institute, 1 Midland Road, London NW1 1AT, UK; 2 Institute of Structural and Molecular Biology, University College London, London WC1E 6XA, UK; 3 Structural Biology Science Technology Platform, The Francis Crick Institute, 1 Midland Road, London, NW1 1AT, UK

## Abstract

The multi-protein complex WRAD, formed by WDR5, RbBP5, Ash2L and Dpy30, binds to the MLL SET domain to stabilize the catalytically active conformation required for histone H3K4 methylation. In addition, the WRAD complex contributes to the targeting of the activated complex to specific sites on chromatin. RbBP5 is central to MLL catalytic activation, by making critical contacts with the other members of the complex. Interestingly its only major structural domain, a canonical WD40 repeat β-propeller, is not implicated in this function. Here, we present the structure of the RbBP5 β-propeller domain revealing a distinct, feature rich surface, dominated by clusters of Arginine residues. Our nuclear magnetic resonance binding data supports the hypothesis that in addition to the role of RbBP5 in catalytic activation, its β-propeller domain is a platform for the recruitment of the MLL complexes to chromatin targets through its direct interaction with nucleic acids.

## INTRODUCTION

The novel motifs generated by methylation of histone lysine residues recruit effector proteins through selective recognition domains and therefore drive the cells gene expression program (1,2). This process must be tightly controlled, which is reflected in the complexity of the trithorax and polycomb methyltransferase complexes that regulate gene activation and repression through histone H3 lysine-4 and lysine-27 methylation respectively (3,4). In addition to their catalytic domains, these complexes contain components that allosterically regulate enzyme activity and others that precisely target the enzyme activity to specific genes (5,6). In higher organisms, histone H3 lysine-4 (H3K4) methylation is carried out by a family of six large multi-domain enzymes, the Mixed Lineage Leukemia (MLL) family. These enzymes share a distinctive C-terminal catalytic SET domain, which is activated by binding to a conserved four-member complex, termed WRAD (7,8). WRAD consists of two β-propeller domain proteins, WDR5 and RbBP5, the SPRY/DNA binding domain protein Ash2L and Dpy30 (9). Of these, WDR5, RbBP5 and Ash2L have been shown to be essential for catalytic activation and are thought to stabilize the active conformation of the SET domain (10,11). Dpy30 may further modulate this activity although the molecular details remain to be elucidated (12).

The MLL family are large proteins, with the major part of the sequence composed of many so-called reader domains, linked by regions of predicted low structural complexity. The function of the reader domains is to target the enzyme by recognition of chromatin or other factors (13). For example, the PHD3-Bromo cassette of MLL1, binds to its own product—methylated H3K4, and facilitates the spread of the activating mark (14). Members of the WRAD complex have also been shown to bind directly to factors that target MLL. For example, the WDR5 β-propeller can bind to unmodified histone H3 (15), Ash2L binds directly to the transcription factors Mef2 and Sox2, and Dpy30 to Oct4 (16,17). In addition to these protein: protein interactions there is mounting evidence of lncRNA-mediated MLL recruitment to specific loci in chromosomes for gene activation (18–20). The details of these interactions are uncharacterized, but one RNA binding interface has been identified on the WDR5 β-propeller and confirmed by mutation of key residues that disrupted normal H3K4 methylation pathways in cells (21). In this manuscript we explore the potential of the RbBP5 β-propeller as another site of RNA interaction.

**Figure 1.**
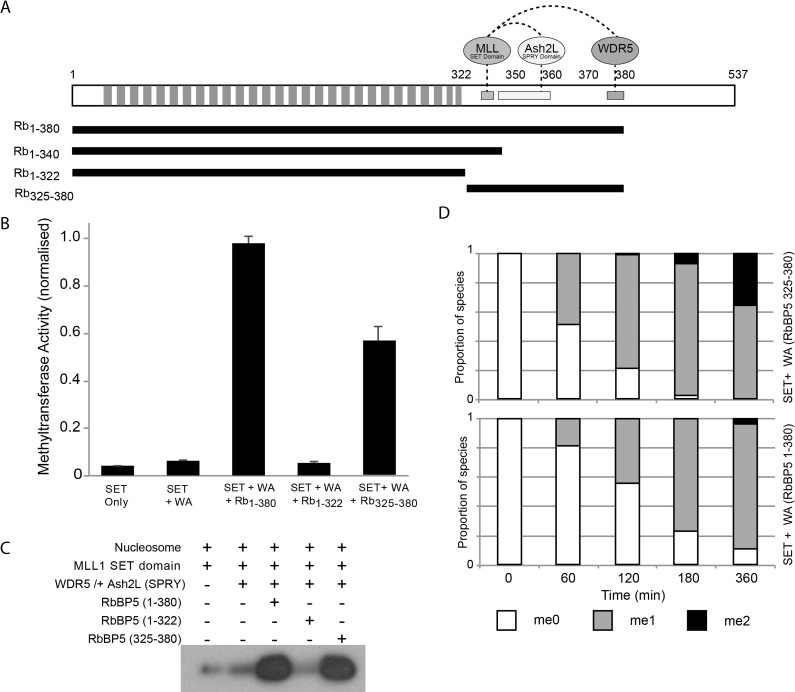
RbBP5 architecture and activity. (**A**) Schematic representation of the RbBP5 protein. The WD40 repeats are indicated along with the three regions shown to interact with the MLL1 SET domain, Ash2L SPRY domain and WDR5. The bars beneath represent the constructs used in the current study. (**B**) Normalized *in vitro* methyltransferase assay with H3 peptide substrate for the MLL1 (SET domain) + WDR5 + Ash2L (SPRY domain) with different RbBP5 constructs showing that only the RbBP5 340–380 region is essential for activity. (**C**) *In vitro* methyltransferase assay with recombinant mononucleosome substrate (147 bp DNA), using a histone H3K4 monomethyl antibody to follow activity. The loading control gel, stained with Coomassie Blue, is presented in Supplementary Figure S1. (**D**) Methyltransferase assay with MLL1 (SET domain) + WDR5 + Ash2L (SPRY domain) with RbBP5 construct plus and minus β propeller analysed by MALDI TOF mass spectrometry.

The amino terminus of RbBP5, residues 1–325, is composed of a series of canonical WD40 repeats that are predicted to form a seven bladed β-propeller domain, which is the major structural feature of the protein (Figure 1A). However, it is the region immediately following this domain, residues 340–380, that stimulates methyltransferase activity through cooperative interactions with the other members of WRAD complex and the MLL SET domain. Specifically, the region RbBP5_344–360_ binds to Ash2L (22), RbBP5_372–381_ to WDR5 (23,24) and RbBP5_330–344_ to the MLL SET domain (11). Thus, only a small 40 amino acid region has an ascribed function, and the role of the β-propeller domain and the remaining 150 C-terminal residues of RbBP5 is currently unknown. Here we focus on the RbBP5 β-propeller domain and show that although it is not essential for WRAD-mediated MLL methyltransferase stimulation, it potentially has a role in targeting the MLL complex. We present the first crystal structure of the β-propeller domain and, supported by nuclear magnetic resonance (NMR) spectroscopy, propose a targeting function through binding of nucleic acid.

## MATERIALS AND METHODS

### Protein expression and purification

The mouse *RbBP5* gene (residues 1–380) was cloned into the pOPINS vector for protein production in *Escherichia coli* BL21 (DE3) cells (24). The 5′ end of *RbBP5 gene* was extended with sequences encoding a 6×His-SUMO tag followed by a Precission 3C protease site (L-E-V-L-F-Q-G-P). RbBP5 (1–380) expression was performed in *E. coli* BL21 (DE3) cells, grown in TB media. *RbBP5* (*1–322*) and *RbBP5* (*1–340*) constructs were generated by introducing a stop codon at the respective sites in *RbBP5* (*1–380*) by site-directed mutagenesis. Site directed arginine to glutamic acid mutants were made in the RbBP5 (1–340) construct. Protein production was induced at OD_600_ of 0.8 with 0.2 mM Isopropyl β-D-1-thiogalactopyranoside (IPTG) for 16–20 h at 20°C. Harvested cell pellets were resuspended in 50 mM Tris–HCl (pH 7.4), 150 mM NaCl, 10% glycerol, 0.5 mM tris(2-carboxyethyl)phosphine (TCEP), 25 μg/ml DNaseI, protease inhibitor cocktail (Sigma) and cells were lysed by sonication on ice. Unbroken cells and cell debris were removed by centrifugation at 20 000 rpm (Sorvall SS-34 rotor) for 60 min at 4°C and cleared lysate was loaded onto a Ni^2+^-NTA gravity flow column containing 3 ml resin (Qiagen). The resin was washed with 10 column volumes of buffer containing 20 mM imidazole and incubated with rhinovirus 3C protease overnight at 4°C. Untagged RbBP5 was separated from the 6×His-SUMO tag bound to Ni^2+^-NTA resin by gravity flow. RbBP5 was finally purified by gel filtration (Superdex 75, GE Healthcare) in 50 mM Tris/HCl (pH 7.4), 150 mM NaCl, 0.5 mM TCEP and 10% glycerol and the fractions of the homogenous main peak corresponding to monomeric RbBP5 were pooled. Purified RbBP5 was snap-frozen in liquid nitrogen and stored at −80°C. The RbBP5 (1–340) construct for NMR was expressed in *E. coli* BL21 (DE3) cells in minimal media with ^15^NH_4_Cl_2_ sole as nitrogen source and purified by affinity and size-exclusion chromatography as described above.

### Crystallization

Crystals of RBBP5 (1–380) were obtained using the vapor diffusion method. RbBP5 (1–380) protein was concentrated to 15–20 mg/ml and used for crystallization by sitting-drop method. Initial crystals appeared in 0.1M MES (pH 6.5), 40% polyethylene glycol (PEG) 200 (V/V) (A7 condition of PEGs suite from Qiagen) at 20°C. The optimized condition included a mixture of additives (H3 of Silver Bullet from Hampton Research) to the original condition. Crystals were harvested directly from the drop and flash-frozen in liquid nitrogen.

### Structure determination

Data were collected at the Diamond Light Source (Oxfordshire, UK) on station I02. The reflections were indexed using XDS and reduced/scaled with programs from the CCP4i suite (25). The structure was solved by molecular replacement using the PHASER package using edited coordinates of WDR5. Difference maps were used to rebuild and extend the initial model using the Coot molecular graphics package (26). Iterative cycles of refinement were carried out using REFMAC (27). Structure visualization was carried out in the PyMol Molecular Graphics System (Schrodinger LLC). Electrostatic surfaces were generated using the APBS plugin (28), protein prepared using pdb2pqr (29), map calculated with grid spacing 0.5 and a solvent excluded surface generated. Conservation was potted onto the surface using the AL2CO sequence conservation analysis server (30), using the multiple sequence analysis presented in Supplementary Figure S2. The coordinates and structure factors for the structure have been deposited in the PDB under accession code 5OV3.

### RNA preparation

All RNAs pools were purchased from Thermo Scientific (Dharmacon). Pools were de-protected following the manufacturer instructions and re-suspended in H_2_O.

### NMR spectroscopy

NMR data were acquired using a Bruker NMR spectrometer operating at 800 and 950 MHz. NMR samples consisted of 40–50 μM ^15^N-labeled RbBP5(1–340) alone and in complex with unlabeled RNA at a 1:1 or 1:4 protein:RNA molar ratio, and were prepared in 350 μl of NMR buffer (10 mM Tris–HCl, 75 mM NaCl, pH 7.2 10% D_2_O). RbBP5–RNA interaction measurements by HSQC NMR were performed at 37°C. To measure Arginine-side chains experiments were performed at lower pH (pH 6.9 at room temperature). The scaffold independent analysis (SIA) strategy and analysis was as previously described (31), using a pool of 6mer nucleotides.

### Methyltransferase assays

Methyltransferase assays were performed using peptide substrates based on the histone H3 amino terminal sequence (ARTKQTARKSTGGKAPR-Y). For end-point assays, H3 peptide concentration was 1 mM peptide, 0.5 mM SAM (including 0.625 mM 3H SAM [PerkinElmer]) in an assay buffer of 50 mM HEPES (pH 8), 200 mM NaCl and 0.5 mM TCEP. Following separation of the peptide from cofactor by C18 cartridge purification, the incorporation of 3H-labeled SAM into the peptide was estimated by scintillation counting, as previously described (32). Methyltransferase assays were carried out at 30°C for 60 min with a final enzyme concentration of 10 μM.

Methyltransferase assays with nucleosome substrate were performed in a buffer containing 20 mM HEPES, pH 7.8, 200 mM NaCl, 2 mM dithiothreitol (DTT). Final reagent concentrations were 5 μM MLL1 SET domain construct (containing the WDR5 interacting motif), WDR5, Ash2L and RbBP5 constructs, 100 μM SAM and recombinant mononucleosome substrate with 147 bp containing the 601 positioning sequence, prepared by the salt dialysis method (33). Reactions were incubated at 30**°**C for 2.5 h and stopped by addition of sodium dodecyl sulphate sample buffer. Following SDS-polyacrylamide gel electrophoresis protein was transferred to Immobilon-P^SQ^ transfer membrane (Merck Millipore Ltd.) and blocked with 5% milk solution. Membrane was probed with histone H3K4 monomethyl-specific antibody (ab176877), (Abcam, Cambridge, UK) at 1:10000 dilution in phosphate buffered saline buffer with 0.05% Tween. The secondary antibody was goat anti-rabbit conjugated to HRP at 1:5000 dilution (Promega) and signal detected by autoradiography following incubation with ECL reagent (Merck Millipore Ltd).

MALDI-TOF methyltransferase assays were performed to examine the reaction products of the methyltransferase reaction with the H3 peptide at specific time points as described previously (34). The reaction mixture contained 10 μM enzyme (MLL1 SET domain containing Win motif, WDR5, Ash2L_SPRY and RbBP5 1–380 or 325–380), 200 μM SAM and 50 μM unmodified H3 peptide at 30°C for up to 6 h. At various time points, aliquots of the reaction were quenched by the addition of an equal volume of 1% trifluoroacetic acid. Samples were diluted in a 1:5 ratio with α-cyano-4-hydroxycinnamic acid and analyzed on a Bruker AutoFlex mass spectrometer (Bruker) in reflectron mode. The reactions were performed in triplicate, and the proportion of methyl species at each time point calculated by combining these multiple measurements.

### Binding measurements

HPLC purified 14-mer DNA/RNA (GCATAGGTTCGATC-6FAM/GCAUAGGUUCGAUC-6FAM) labeled with 6-FAM fluorescein molecules were purchased from Sigma. Equilibrium dissociation constants for the interaction of RbBP5 (P) with FAM–RNA (L) to form the complex RbBP5:RNA (PL) were determined using anisotropy titrations. For any mixture of RbBP5 and FAM–RNA the observed anisotropy (*r*_OBS_) is given by:

(1) \begin{equation*}{r_{{\rm OBS}}} = \frac{{\alpha {r_{{\rm PL}}}[{\rm PL}] + {r_L}[L]}}{{\alpha [{\rm PL}] + [L]}}\end{equation*}

where *r*_PL_ and *r*_L_ are the anisotropies of the complex and the free nucleic acid, and *α* ( = 1 in this case) is the fluorescence intensity of the complex divided by that of the free nucleic acid. The data were analyzed using non-linear least-squares fits to equation (1) with *r*_PL_, *r*_L_ and the dissociation constant (*K*_d.L_) as variables, and PL and *L* calculated in the usual way:

\begin{eqnarray*} \begin{array}{*{20}{l}} {[{\rm{PL}}]}\\ { = \frac{{\left( {{{{K}}_{{\rm{d,L}}}} + [{{{P}}_0}{\rm{] }} + {\rm{ [}}{{{L}}_{\rm{0}}}]} \right) - \sqrt {{{\left( {{{{K}}_{{\rm{d,L}}}} + [{{{P}}_0}] + [{{{L}}_0}]} \right)}^2} - 4[{{{P}}_0}][{{{L}}_0}]} }}{2}\,\,\&\,\, [{{L}}] = [{{{L}}_0}] - [{\rm{PL}}]} \end{array} \end{eqnarray*}

where the subscript 0 indicates the total concentration of the species.

All anisotropy titrations were performed at 20°C using a Jasco FP-8500 fluorimeter. The buffer (50 mM Tris pH 7.4, 150 mM NaCl, 0.5 mM DTT). Measurements with dsRNA and dsDNA were performed in identical conditions. Measurements with RbBP5 mutants were performed as above with dsRNA.

## RESULTS

### The RbBP5 β-propeller domain is not essential for catalysis

The RbBP5 region covering residues 340–380, immediately C-terminal to the β-propeller domain, has been shown to bind to other components of the catalytically active MLL complex and to be essential for methylation (11). We were first interested to determine if the β-propeller domain contributes to the activity of the assembled complex. We therefore compared the *in vitro* methyltransferase activity with H3 peptide substrate for the MLL1 SET domain reconstituted with Ash2L/WDR5 and different RbBP5 constructs (Figure 1). As expected the MLL1 SET domain alone exhibited negligible methyltransferase activity, which only modestly increased with addition of WDR5/Ash2L (SPRY) (Figure 1B). However, the further addition of RbBP5_1–380_ strongly stimulated methylation confirming the integral role that this protein has in assembly of the catalytic core. In line with the observation of Li *et al.* (11), a RbBP5_325–380_ construct also significantly stimulated activity, confirming that the elements responsible for assembling the complex and stabilizing the active SET domain conformation, are located in this region. However, interestingly, we observed that the RbBP5_1–322_ construct, in combination with WDR5/Ash2L (SPRY), did not stimulate the overall activity of MLL1, indicating that the β-propeller domain does not contribute significantly to the catalytic activation mechanism. The slightly higher activity observed for RbBP5_1–380_ compared to RbBP5_325–380_ may arise due to better construct stability or improved complex integrity. The equivalent methyltransferase assay, but using a recombinant mononucleosome (147 bp DNA) substrate, showed a similar pattern of MLL activation, i.e. activity was dependent on the presence of the RbBP5_325–380_ region, but the β-propeller was not required (Figure 1C). This suggests that the β-propeller is neither essential for assembly of the catalytically active complex or for recognition of core nucleosome.

The potency of histone methylation as an epigenetic signaling mark lies in the high degree of specificity that recognition domains exhibit for the number of methyl groups on the methylated lysine. We were therefore interested to determine if the presence of the β-propeller domain had an effect on the final reaction product. We compared the product of the methyltransferase reaction with peptide substrate using MALDI TOF analysis for the MLL1 (SET)/WDR5/Ash2L (SPRY) complex with RbBP5_1–380_ or RbBP5_325–380_ constructs. In both cases the H3K4_me1_ and to a lesser extent H3K4_me2_ product were readily detected, consistent with the previously reported activity of the reconstituted complex with peptide substrate (10,34) (Figure 1D). The amount of me_2_ product produced by the RbBP5_325–380_ construct was slightly lower than the longer construct, which may be attributed to a modest effect on the overall stability of the complex, but these data indicate that the RbBP5 β-propeller does not significantly contribute to catalytic activity and suggests that it has a different role.

### Structure of the RbBP5 β-propeller

**Figure 2.**
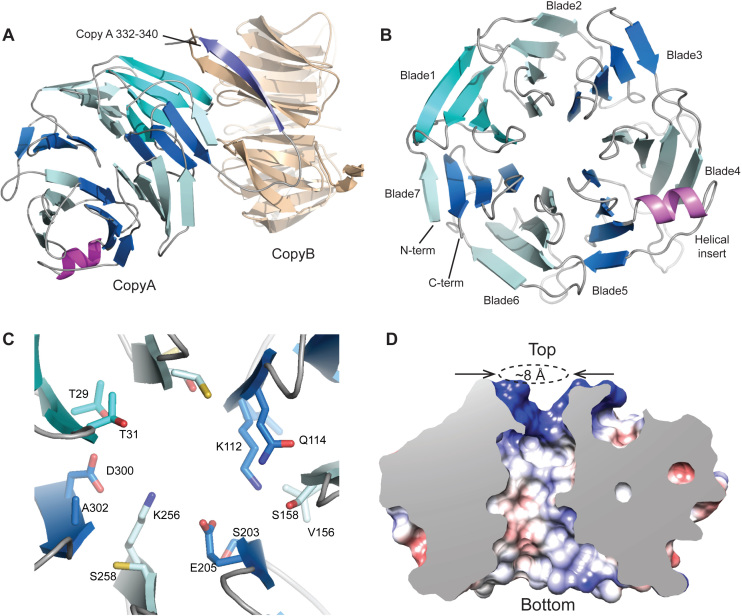
Structure of the RbBP5 β-propeller domain. (**A**) Cartoon representation of the RbBP5 asymmetric unit. The blades of the Copy A β-propeller are colored in alternating shades of blue. Copy B is colored in beige. The Copy A residues 332–340 displace the N-terminal strand in Copy B to form a crystallographic dimer. (**B**) Top view of the RbBP5 β-propeller showing a canonical arrangement of seven 4-stranded blades. A helical insert (pink) is located at the interface of blades 4 and 5. (**C**) The RbBP5 central channel is formed from largely polar residues. (**D**) Cut-through of RbBP5 (electrostatic surface representation) revealing the wide polar channel.

To obtain a better understanding of the characteristics of the WD40-repeat rich region, we determined the crystal structure of mouse RbBP5. After screening a wide range of constructs, diffracting crystals of the RbBP5_1–380_ construct were obtained, which contains both the β-propeller and the regions known to interact with other members of the MLL complex. The structure was determined by molecular replacement, for details see ‘Materials and Methods’ section, and refined to a resolution of 2.5Å. The asymmetric unit contains two copies of the construct (Figure 2A), and data collection and refinement statistics are presented in Table 1. The β-propeller domain structure is basically consistent between both copies, but the 325–380 region is largely disordered and varies significantly. Differences arise both in the course it takes with respect to the propeller, and to the number of residues which could be built into interpretable electron density. This can be interpreted as indicative of an unstructured region, but one that adopts a conformation induced by the interaction with its binding partners when assembled into the complex (11,22,24). The two copies of the β-propeller are linked in the crystal structure due to a strand swap which disrupts the seventh blade of the copy B propeller. The copy A residues 332–340 pack as a parallel β-strand against the copy B strand, displacing the N-terminal residues 18–22, which completes the propeller blade through the canonical interaction. This induces the third strand in the blade to extend by four residues to Val 326. This organisation is most likely a crystallographic artefact, notably we observed no dimerization in solution either during purification or by Multi-Angle Laser Light Scattering (Supplementary Figure S3) analysis. In the analysis below we focus on the β-propeller of copy A, which represents the physiological form of the propeller.

**Table 1.** Data collection and refinement statistics

	5OV3
Data collection	
Wavelength (Å)	0.9795
Space group	P2_1_2_1_2_1_
Cell dimensions	
*a, b, c* (Å)	56.81, 71.9, 178.27
α, β, γ (°)	90.00, 90.00, 90.00
Resolution (Å)	71.90–2.45 (2.58–2.45)
*R*_merge_	0.10 (1.26)
*R_pim_*	0.043 (0.59)
*I* / σ*I*	12.7 (1.6)
CC_1/2_ (%)	99.8 (64.9)
Completeness (%)	99.8(99.2)
Redundancy	6.3 (5.4)
**Refinement**	
Resolution (Å)	66.7–2.45
No. reflections	27559
*R*_work_/*R*_free_	0.196/0.271
No. atoms	
Protein	5093
Ligand (PEGs)	23
*B*-factors	
Protein	59.3
R.m.s. deviations	
Bond lengths (Å)	0.009
Bond angles (°)	1.041
Ramachandran Outliers	1.39 %
Rotamer Outliers	2.3 %

RbBP5 forms a canonical seven bladed WD40 repeat β-propeller in which the seventh blade is formed from three anti-parallel strands from the C-terminal region of the domain and is completed with a strand originating from the N-terminus of the domain (35) (Figure 2B). Between the third and fourth strands of the 5^th^ propeller blade there is an insert which includes a short α-helix, which lies on one face of the propeller toward the rim (Figure 2B). Helical inserts are common in WD40 repeat proteins, and have been observed in other chromatin associated β-propellers, such as RbAp48 and embryonic ectoderm development (EED), where they are associated with protein interactions. For example, in RbAp48 the helical insert on the side of the propeller interacts with Histone H4 (36,37).

A conspicuous feature of the RbBP5 β-propeller, which appears to be quite distinct from other WD40 proteins in chromatin modification complexes such as WDR5 in MLL and EED and RbAp48 in PRC2, is that the central axis is not formed from bulky aromatic side chains, but instead polar residues dominate the propeller axis (Figure 2C). This creates a much more open solvent filled channel of about 8Å diameter that links the two faces (Figure 2D). The top channel edge is strongly basic, and at the ‘bottom’ the channel flares out creating a concave feature on that face of the propeller. Potentially these patches could mediate targeting interactions critical to the function of the MLL complex.

**Figure 3.**
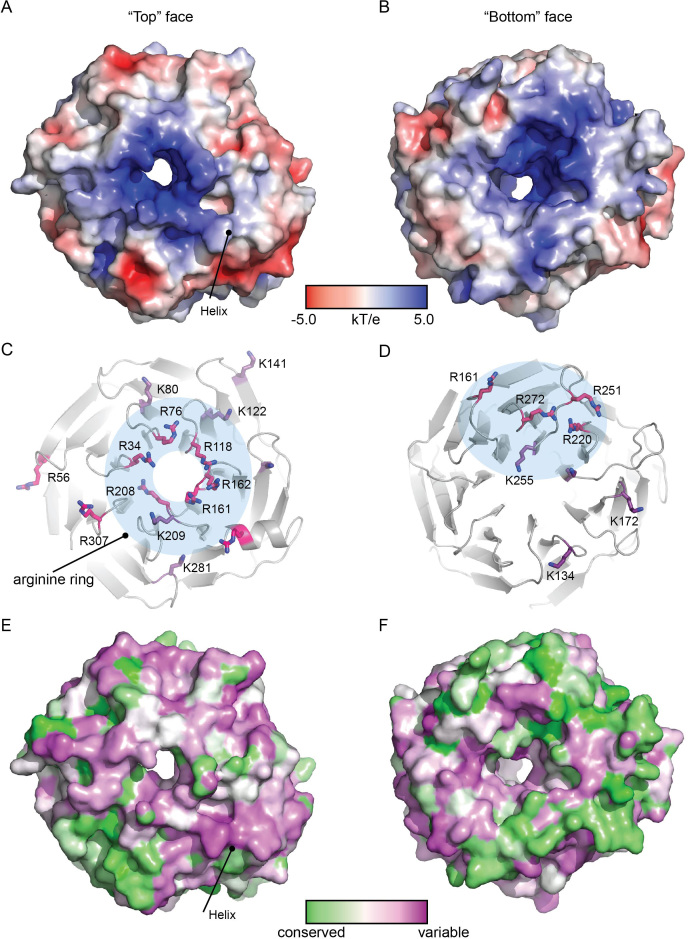
Surface features of RbBP5. (**A, C** and **E**) Views of the ‘top’ of the RbBP5 β-propeller, showing (**A**) Electrostatic surface of the ‘top’ face of the β-propeller. The position of the helical insert is indicated. (**C**) Cartoon representation of the ‘top’ face of the β-propeller. The Arginine (magenta) and Lysine (purple) side chains are shown, with the Arginine ring feature indicated by a blue ring. (**E**) Sequence conservation mapped onto the top face of the β-propeller (Gradient: green (conserved) to magenta (variable)). (**B, D** and **F**) Views of the ‘bottom’ surface of the propeller; (**B**) Electrostatic surface of the ‘bottom’ face of the β-propeller. (**D**) Bottom view of the propeller showing the Arginine (magenta) and lysine (purple) side chains. (**F**) Sequence conservation mapped onto the ‘bottom’ face of the β-propeller.

Both faces of the RbBP5 β-propeller feature prominent basic patches (Figure 3A and B). On the ‘top’ face, defined as the face containing the α-helical insert, the propeller axis has a complete ring of basic side-chains, which extends into a basic groove between blades six and seven, and so reaches toward the edge of the propeller. These features arise from Arginine and Lysine side-chains, with the central ring feature consisting of residues Arg34, 76, 118, 161, 162, 208 and Lys209 located on six of the seven blades (Figure 3C). These residues are distributed across the primary sequence of the domain and do not form a discrete motif; the residues are conserved in the RbBP5 homologs from higher organisms, only partially in *Neurospora*, but not at all in yeast species (Figure 3C and E; Supplementary Figure S2). This suggests that this feature may have evolved in multi-cellular organisms, which is suggestive of a potential role in cell lineage targeting, perhaps during developmental processes. The bottom face of the β-propeller is also characterized by a basic patch (Figure 3B), which extends from one side of the large cavity that surrounds the central axis to the edge of the face (Figure 3D). These residues, Arg220, 251 272, and 294, and Lys 255, which form the basic batch on the bottom surface are more broadly conserved across species, (Figure 3F and Supplemental Figure S2). In general, the bottom face of the propeller is better conserved than the top (Figure 3E and F), which may indicate that it could mediate an interaction that is fundamental to basic function.

Overall, the principal feature of the RbBP5 β-propeller surface, is that it is predominantly polar, both at the open central axis and the basic patches that characterize both top and bottom faces of the propeller. We were therefore interested to identify binding partners for this domain.

### RbBP5 binds nucleic acids using an Arginine-rich surface

Given that the RbBP5 β-propeller does not have a role in modulating the activity of MLL1 we hypothesized that this domain is most likely a platform for binding to unidentified partners involved in targeting the complex to chromatin features or to specific genes. We explored whether the domain binds to histone tails using a biotin tagged RbBP5 β-propeller construct to probe a histone peptide array containing duplicates of 384 of the common epigenetic modifications (Active Motif), but no hits were identified using this strategy (data not shown). Next, we reasoned that given the basic characteristic of the features identified on the surface of the β-propeller, and the increasing evidence of involvement of long non-coding RNA in targeting MLL1 (21,38), it was plausible that the domain may recognize nucleic acid rather than a protein partner.

**Figure 4.**
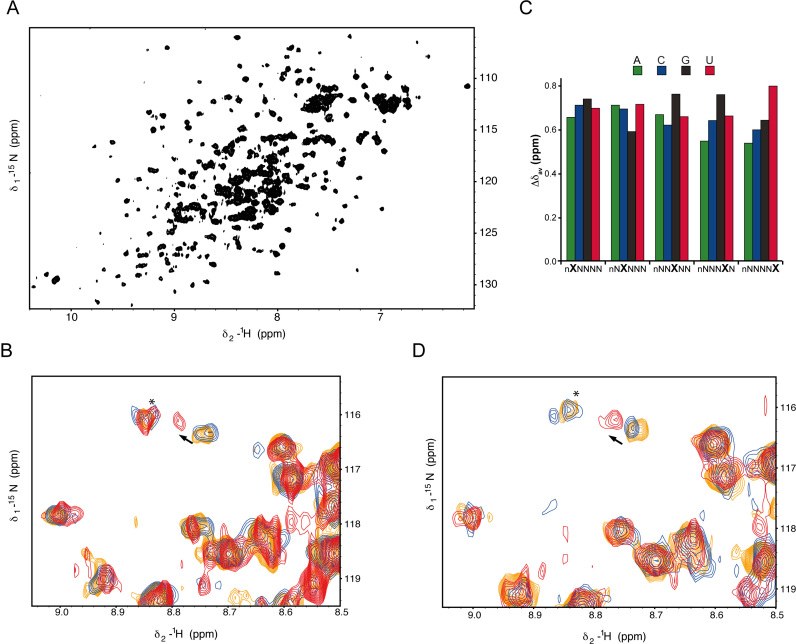
Analysis of RNA binding to the RbBP5 β-propeller. (**A**) ^1^H^15^N HSQC spectrum of the RbBP5_1–340_ construct showing well-dispersed resonances. (**B**) Titration of a randomized RNA 7-mer oligo nucleotide induces selective resonance shifts in the ^1^H^15^N HSQC spectrum. Blue apo RbBP5, orange 1:1, red 4:1 RNA:protein. (**C**) Scaffold Independent Analysis showing the preference of RbBP5 for each nucleobase at five consecutive positions in the oligonucleotide. (**D**) Titration of a sequence optimised RNA 7-mer induces selective resonance shifts in the^1^H^15^N HSQC NMRspectrum of RbBP5.

To test RbBP5′s capability to bind nucleic acids we used an NMR spectroscopy approach. NMR can deliver information on protein-ligand interactions covering a broad range of affinities and is ideal for such a *de novo* exploration. The ^1^H^15^N HSQC spectrum of the RbBP5_1–340_ construct is of a good quality considering the protein's size. The resonances are well dispersed and the peaks of relatively uniform intensity confirm that the protein is folded, soluble and monomeric (Figure 4A). As no nucleic acid target of the protein is known, we tested RbBP5 binding to both ssRNA, dsRNA and to dsDNA. We first titrated the protein with a pool of short 7-nucleobase RNA oligos with random sequence (7N-RNA). Titration of 7N-RNA shifted a subset of resonances, indicating an interaction is taking place with a discrete selection of RbBP5 residues (Figure 4B). The resonances are in fast exchange on a chemical shift timescale, which is consistent with a dissociation constant in the micromolar range. Further, the small number of peaks changing chemical shift indicates that the contacts with backbone moieties are not extensive and that it is likely that no major structural rearrangement is taking place in the RbBP5 upon associating with RNA. This is consistent with a surface feature such as those formed by the arginine side-chains.

Next, we examined whether the interaction with ssRNA is sequence specific using ‘SIA’ (31). This method provides the nucleobase preference of a protein domain for the different positions of the bound RNA, and performs best at intermediate to low affinity and with transient protein–RNA interactions. SIA was used to test the preference for each nucleobase at five consecutive positions of a bound oligonucleotide (Figure 4C). The SIA results indicate that RbBP5 has relatively little sequence specificity, with a very modest preference for G and U. Consistently, an SIA optimized RNA oligo (UAGGUUC) based on this preference bound only marginally stronger to the RbBP5 β-propeller than the randomized oligo pool (Figure 4B and D).

**Figure 5.**
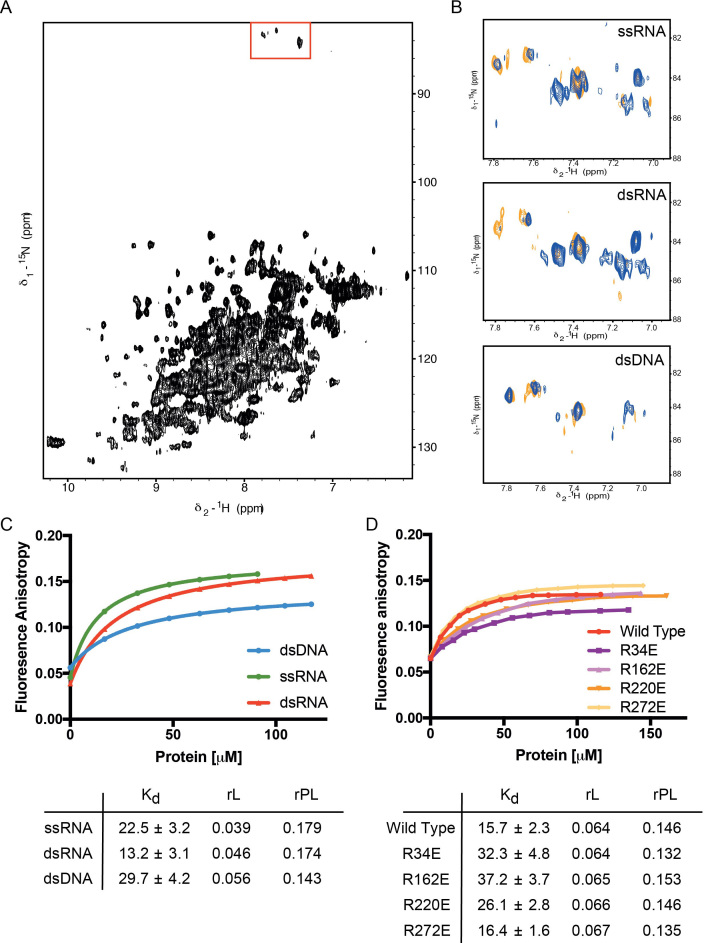
RbBP5 binding to nucleic acids. (**A**) ^1^H^15^N HSQC spectrum of RbBP5_1–340_ construct optimized for the detection of arginine side chain moieties, indicated by red box at top. (**B**) Spectrum of RbBP5 free and titrated with ssRNA, dsRNA and dsDNA (orange apo RbBP5, blue 4:1 NA:protein). (**C**) Fluorescence anisotropy measurements of RbBP5_1–340_ binding to fluorescein labeled nucleic acid. (**D**) Fluorescence anisotropy measurements of acidic mutants of RbBP5_1–340_ binding to fluorescein labeled dsRNA.

Given that only a small number of RbBP5 resonance shifts were observed upon addition of RNA and that the nucleobase preference exhibited was very limited, we wondered whether the RbBP5–RNA interaction was mediated with low specificity by a subset of the Arginine side chains on the β-propeller surface. In order to test this, we recorded ^1^H^15^N HSQC experiments optimized to capture changes in the resonances of the Arginine side chain resonances (Figure 5A). RNA titration results in the shift of a few of the existing resonances and in the appearance of a number of new resonances. The strong effect of the RNA on the linewidth of selected Arginine side chains is consistent with the RNA interacting with the charged side chains of a group of Arginines on the RbBP5 surface and slowing the exchange of the ^15^N_attached protons with the bulk water.

Next, we tested whether RbBP5 preferred DNA to RNA or if a structural preference exists for ssRNA versus dsRNA. We recorded Arginine-optimized NMR experiments using 14-mer ssRNA, dsRNA and dsDNA molecules of the same sequence (except uracil were changed for thymine in DNA) and length to titrate against RbBP5_1–340_ (Figure 5B). The effect was strongest when titrating dsRNA and weakest when titrating dsDNA, with the ssRNA having an intermediate effect. To determine whether this effect can be correlated to a difference in binding affinity, we performed a fluorescence anisotropy assay to measure equilibrium dissociation constants for the binding of 6-carboxy fluorescein labeled 14-mer oligos of ssRNA, dsRNA or dsDNA of identical sequence (Figure 5C). The anisotropy measurements confirmed binding of all three nucleic acid species, consistent with the NMR titrations. Further, also consistent with the NMR titration RbBP5 showed a modest preference for double stranded RNA (13.2 μM), over single stranded RNA (22.5 μM) and double stranded DNA (29.7 μM). Overall our data indicate that RbBP5 recognize dsRNA with moderate affinity and structure specificity and confirm that the interaction is mediated by Arginine side chains.

Given that there are basic patches on both faces of the β-propeller, we were interested if nucleic acid binding was limited to either face. Two candidate side chains were selected from each patch and mutated to acidic residues. The fluorescence anisotropy binding measurement was repeated with dsRNA for wild-type β-propeller and the four mutant proteins (Figure 5D). Only modest changes in affinity were observed for the single point mutants, which is consistent with a broad interface. Both upper face mutants (R34E and R162E) exhibited a greater than 2-fold loss of affinity, whereas only one of the bottom face mutants (R220E) differed significantly from the wild-type. This limited evidence favors a model in which the upper Arginine ring promotes the interaction, but does not preclude the possibility that with a larger nucleic acid substrate an interface involving both surfaces may form, or that the faces may be involved in independent interactions.

## DISCUSSION

The MLL histone methylation complexes have two distinct functions; one is the targeting function, and this is the role of the major part of the MLL proteins themselves, as they are essentially a series of recognition domains linked by regions of predicted unstructured sequence. The other is the catalytic function, comprising the complex formed by the extreme C-terminal region of the MLLs and the four WRAD complex proteins (9). The role of the RbBP5 region (residues 330–380) in organizing the activated complex structure by linking Ash2L, WDR5 and the SET domain is well described (11,22,24,39). Our *in vitro* methylation data show that the main structural element of RbBP5, the β-propeller domain, does not play an integral role in MLL complex assembly or catalytic activation. This suggests that the RbBP5 β-propeller may have a role in targeting the MLL/WRAD activity or even a function outside of the MLL complex.

The other β-propeller protein in the WRAD complex, WDR5, has been shown to be a component of multi-protein complexes independently of the other WRAD proteins or MLL (40,41). For example, WDR5 is an integral component of the histone acetyltransferase complexes NSL/MOF where it binds to the NSL1 subunit (42) and the ATAC complex (43). WDR5 is ubiquitously expressed and its high abundance in the cytoplasm also suggests that it has roles beyond chromatin modification complexes (44). There are fewer examples suggesting that RbBP5 has such a varied function. However, recent proteomic studies point to several chromatin associated proteins that may potentially participate in RbBP5 interactions in an MLL context, including Chromo Helicase Domain 8 (45), Cfp1 (46), OGT (47) and Cul4A-DDB1 (48), but further studies will be required to determine if these are direct contacts.

Given that it does not appear to have a direct role in formation of the catalytic core, the most likely function of the RbBP5 β-propeller could be in recruitment of MLL to specific sites via modified chromatin. However, using a histone modification peptide array consisting of a comprehensive range of typical posttranslational modifications associated with epigenetic signaling, we were unable to identify any hits. It should be noted that this does not preclude that RbBP5 could bind to histone tails in the context of an extended nucleosome array with linker DNA or to an untested combination of modifications. However, the prominent features on the RbBP5 surface are basic patches, and it is unlikely that the generally basic histone tails are interaction partners.

Arginine ring or Arginine patch features occur in a range of proteins, and in a number of cases have been shown to interact with nucleic acid rather than proteins (49,50). For example, the DDB2 β-propeller has a surface patch of basic residues that bind to DNA through contacts with the phosphate backbone (51). This encouraged us to investigate if we could detect direct binding of RNA to the RbBP5 β-propeller. Our NMR and fluorescence anisotropy measurements show that RbBP5 binds to nucleic acid via selected Arginine side chains, consistent with the binding taking place in the large positive patch that surrounds one end of the domains channel. Further, the experiments show a modest preference of the protein for double stranded RNA over single stranded RNA. This would be consistent with a lncRNA, which are often structured, but as only modest variations in affinity were observed with the different types of nucleic acid, more experiments will be required to identify the physiological binding partner or partners.

There is growing evidence that at least part of the targeting function of chromatin regulatory protein complexes such as trithorax and polycomb group during development is mediated by lncRNAs (52,53). The list of lncRNAs with a putative role in MLL mediated H3K4 methylation is expanding, and examples include HOTTIP, Mistral, HoxBlinc and SnoRNA/116HG (20,54–57). Although the site of interaction of many of these lncRNAs is unknown, WDR5 has been implicated in some interactions (21). However, there is some evidence supporting direct interaction between RbBP5 and RNA. For example, depletion of SnoRNA/116HG, a lncRNA, is linked to Prader-Willi syndrome. In a co-precipitation experiment with RbBP5 used as bait protein *116HG* RNA was precipitated in conditions that favored formation of RNA secondary structure (18). RbBP5 has been identified as an RNA interacting protein in two recent large-scale screens for novel RNA binding proteins (58,59). In one experiment RNA co-precipitated with a peptide that corresponded to RbBP5_61–76_. Notably, this region incorporates one of the Arginines associated with one of the basic patches identified in our structural analysis.

Our structural analysis has revealed that the RbBP5 β-propeller has a feature rich surface that is likely to mediate as yet unknown binding interactions. It is also likely that, like its WRAD complex partner WDR5, the RbBP5 β-propeller may mediate interactions with multiple partners in different contexts. However, our binding analysis leaves open the possibility that lncRNA is one potential partner.

## DATA AVAILABILITY

The coordinates and structure factors for the structure have been deposited in the PDB under accession code 5OV3.

## Supplementary Material

Supplementary Data
