# Supplementary material for: The structure of the RbBP5 β-propeller domain reveals a surface with potential nucleic acid binding sites
Source: Nucleic Acids Res. 2018 Mar 21;46(7):3802–12. doi: 10.1093/nar/gky199 (PMC6283417; doi:10.1093/nar/gky199)
Supplement: Supplementary Data [file gky199_supplemental_files.pdf]

The structure of the RbBP5 Beta-Propeller domain reveals a surface with potential nucleic acid binding sites

Ansumali Mittal[1,3], Fruzsina Hobor[2,4], Ying Zhang, Stephen R. Martin, Steven J. Gamblin[1], Andres Ramos[2] and Jon R. Wilson[1,*]

[1]The Francis Crick Institute, 1 Midland Road, London NW1 1AT, UK.

[2]Institute of Structural and Molecular Biology, University College London, London, WC1E 6XA.

[3]Current address: Tufts University School of Medicine,136 Harrison Avenue, Boston, MA 02111. USA

[4]Current address: Faculty of Biological Sciences, University of Leeds, Leeds LS2 9JT. UK

[5]Structural Biology Science Technology Platform, The Francis Crick Institute.

[*]Email: jon.wilson@crick.ac.uk

Supplemental Files

Supplementary Figure 1: Loading control for Methyltransferase gel assay

Following the methyltransferase assay with nucleosome substrate (Figure 1C), the samples were split and equal volumes loaded onto two halves of the gel (NuPAGE 4-10 %, Mes buffer (Thermo Life Sciences)) for electrophoresis. One half was blotted as described in the Methods section, the second half was stained with InstantBlue (Expedion) Coomassie stain and presented to show loading. The identity of each protein band is as indicated.

Supplementary Figure 2: Sequence analysis of RbBP5 proteins.

Multiple sequence alignment of RbBP5 sequences for a range of species across the $RbBP5_{1-380}$ construct. Secondary structure elements from the current structure are indicated above the sequence and coloured according to Figure 2. Axis surface residues are indicated in red highlighting. Top surface Arginine ring residues are indicated by purple highlighting and bottom surface Arginine patch residues are indicated by magenta highlighting. Known interactions at the C-terminus of the construct are indicated in gray.

Supplementary Figure 3: SEC-MALS of $RbBP5_{1-365}$

The $RbBP5_{1-365}$ construct was expressed, tag cleaved and purified as described in the methods section of the main manuscript. $RbBP5_{1-365}$ at 2 mg/ml was loaded onto a Superdex 200 Increase GL 10/300 column (GE Healthcare) at 1 ml/min. Buffer 50 mM Tris, PH7.5, 100 mM NaCL, 0.5 mM TCEP , 1% sodium azide. Multi-Angle Light Scattering analysis (Astra Software, Wyatt Technology) indicated that the RbBP5 construct eluted on the gel filtration column as a monomeric monodisperse peak.

Supplementary Figure 1

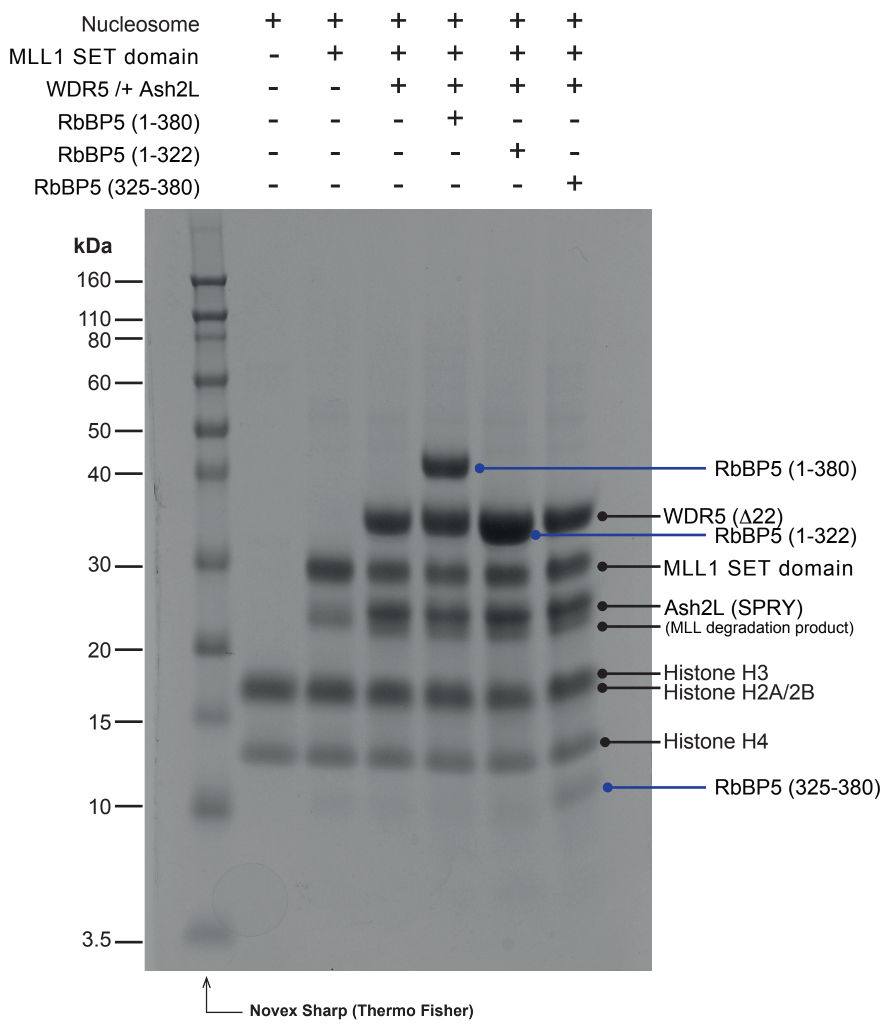

Supplementary Figure 2

```
Human          MNLELLESFG--QNYPEEADGTLDCISM-ALTCTFNRWGTLLAVGCNDGRIVIWDFLTR-GI---AKIISAH--IHPV
Mouse          MNLELLESFG--QNYPEEADGTLDCISM-ALTCTFNRWGTLLAVGCNDGRIVIWDFLTR-GI---AKIISAH--IHPV
Zebrafish      MNLELLESFG--QNYPEEADGTLDCISM-ALTCTFNRWGTLLAVGCNDGRIVIWDFLTR-GI---AKIISAH--IHPV
Drosophila     MNLELLESFG--QNYPEEFDGSLDCISL-AVTCAFNKYGTLLAVGCNDGRIVIWDFLTR-GI---AKIISAH--VHPV
Worm           MVEILDRTYG--AQFPEELECHLDLQNASANCCKFNRWGSIVAVGCTDGRVLIYDFMTR-NI---ARTFSAH--CLPV
Neurospora     MNLLLAEDYL-LQDYPEHITNTIRSG--HSTCVRFNRTGDFLASGRVDGTVVIWDLETM-GV---ARKLRGH--SKNI
Fission Yeast  MNLELLDPFS-IPDYPEALTTTLKHG--HATSIRFSTNGYHLASGLVNGSVVIWDLSTF-SV---SRVLTGH--TRAI
Budding Yeast  MNILLQDPFAVLKEHPEKLTHTIENPL-RTECLQFSPCGDYLALGCANGALVIYDMDTFRPICVPGNMLGAH--VRPI

Human          CSLCWSRDGHKLVSASTDNIVSQWDVLSGDC-DQRFRFPSPILKVQYHPRDQ---NKVLVCPMK----------SAPV
Mouse          CSLCWSRDGHKLVSASTDNIVSQWDVLSGDC-DQRFRFPSPILKVQYHPRDQ---NKVLVCPMK----------SAPV
Zebrafish      CSLCWSRDGHKLVSASTDNIVSQWDVLTGDC-DQRFRFPSPILKLQYHPRDM---NKVLVCPMK----------SAPV
Drosophila     CSLSWTRNGHKLLSASTDNNVCIWDVLTGEL-EHKYRFPSPVLKVQFDPRND---NRLLVCPMR----------YAAV
Worm           SCLSWSRDGRKLLTSSADNSIAMFDVLAGTL-LHRIRFNSMVTFAMFHPRND---NKAIVLQVN----------KQPT
Neurospora     TSLSWSRCGRYLLSACQGWKAILWDLQDGSK-YCEVRFRAPVYGAELHPMHH---HQFAAALFE----------EQPM
Fission Yeast  QSVCWSSCDRFLLTASRDWKCILWDLRDGSI-VYQVVLSAPVWSASLHPHKI---NTFVASLLD----------ESPQ
Budding Yeast  TSIAWSPDGRLLLTSSRDWSIKLWDLSKPSKPLKEIRFDSPIWGCQWLDAKR---RLCVATIFEESDAYVIDFSNDPV

Human          MLTLSDS--KHVVLPVDDD---------SDL------------------NVVASFDRRGEYIYTGNAKGKILVLKTDS
Mouse          MLTLSDS--KHVVLPVDDD---------SDL------------------NVVASFDRRGEYIYTGNAKGKILVLKTDS
Zebrafish      LLTLSDS--KHVVLPVDDD---------SDL------------------NVVAAFDRRGEYIYTGNAKGKILVLNTDT
Drosophila     LVEVG-G--THRCLPLDSD---------GDL------------------NIVASFDRRGKHIYTGNAKGKILVLDVET
Worm           VEQFSPR--IQTVLANDTP--------GSSDE-----------------SASCVSYDRKGKYIIAGTGKGKLLIYNAET
Neurospora     LVDVKES--AQGANPVEVRHILPSVEKRSDDGADTPTKEKHAKEDARHMTTAIVYTATGEHLLAGTTKGRLNIIDATT
Fission Yeast  LIIVDDGIPKHKYLPTNPD---------IDEN-YS-----DRRNRSKHVTLVSFFHPSGEYILSGTSKGWFHVIDAST
Budding Yeast  ASLLSKS--DEKQLSST---------------------------PDHGYVLVCTVHTKHPNIIIVGTSKGWLDFYKFHS

Human          Q---DLVASFRVTTGTSNTTAIKSIEFARKGSCFLINTADRIIRVYDGREILTCGRD---GEPEPMQKLQDLVNRTPW
Mouse          Q---DLVASFRVTTGTSNTTAIKSIEFARKGSCFLINTADRIIRVYDGREILTCGRD---GEPEPMQKLQDLVNRTPW
Zebrafish      Q---DLVASFRVTTGTSNTTAIKSIEFARKGSCFLINTADRIIRVYDGREILTCGRD---GEPEPMQKLQDLVNRTPW
Drosophila     F---EVVASFRIIVGTSSATAVKSIEFARRGDAFLINTSDRVIRVYDSKEIITLGKD---GEPEPIQKLQDLVNKTTW
Worm           L---KCVAWCKQNT----VQQIRQIIVPMKSRFIITNTQDRVIRTYELEDLLH-QRG---QMVEAKYKVLDMVNKAAW
Neurospora     H---KIIYSEKIAG-----GVITTLRLTESGKELLVNAQDRTIRTFKVPDLTSADLDPDTIQIPLEHKFQDLVNRLSW
Fission Yeast  T---KIRSSHRITS-----QSIKQIRLSFCKRFLIFNSTDRVIRTVSIQDL---------DNPEVEHKFQDVVNRLQW
Budding Yeast  LYQTECIHSLKITS-----SNIKHLIVSQNGERLAINCSDRTIRQYEIS-IDDANSA---VELTLEHKYQDVINKLQW

Human          KKCCFSGD-GEYIVAGSA--RQHALYIWEKSIGNLVKILHGTRGELLLDVAWHPVRPIIASIS--SGVVSIWAQNQVE
Mouse          KKCCFSGD-GEYIVAGSA--RQHALYIWEKSIGNLVKILHGTRGELLLDVAWHPVRPIIASIS--SGVVSIWAQNQVE
Zebrafish      KRCCFSGD-GEYIVAGSA--RQHALYIWEKSIGNLVKILHGTRGELLLDVAWHPVRPIIASIS--SGVVSIWAQNQVE
Drosophila     KKCCFSGD-GEYICAGSA--RQHALYIWEKSIGNLVKILHGTKGELLLDVVWHPVRPIIASIS--SGLVSIWAQNQVE
Worm           KNVCTDSD-GLYVCGAST--KAHSLYIWESNTGSLIKILHGNKGEALLDVQWHPTRPIILSIA--QGTVSMWTQAHVE
Neurospora     NHAAFSST-GEYVAASTF--NNHELYIWERGHGSLVRMLEGPKE-EQGVIEWHPHKPLLAACGLETGRINIWSVTSPQ
Fission Yeast  NSCGFSQT-GEFVFATTYQ-MAHAIYVWERGMGSLVKILEGPKE-ELVDVDWHPVFPCVASVGLDSGSIYIWAVEQKE
Budding Yeast  NCILFSNNTAEYLVASTHGSSAHELYIWETTSGTLVRVLEGAEE-ELIDINWDFYSMSIVSNGFESGNVYVWSVVIPP
```

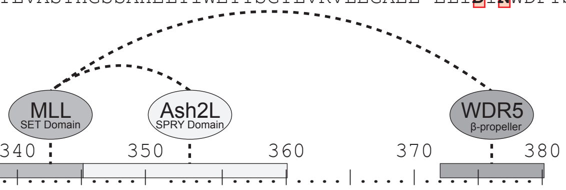


```
Human          NWSAFAPDFKELDENVEYEERESEFDIEDEDKSEPEQTGADAAEDEEVDVTSVD
Mouse          NWSAFAPDFKELDENVEYEERESEFDIEDEDKSEPEQTGADAAEDEEVDVTSVD
Zebrafish      NWSAFAPDFKELDENVEYEERESEFDIEDEDKSEPEQTGADAAEDEEVDVTSVD
Drosophila     NWSAFAPDFKELDENVEYEERESEFDIADEDKSVDLNADAQQDEEIEVDVQKVE
Worm           NWSAFAPEFQELEENEKYIEKENEFDMEDEDADEDMTSKSQDAEDDIIDVVNVR
Neurospora     RWSALAPDFVEVEENVEYIEKEDEFDIHPHEEIQK---RRLDAEDEDVDVLGGG
Fission Yeast  SWSAFAPDFQELEENIEYEEPEDEFDIHDETGKSE-------EEEYFTSVVKIL
Budding Yeast  KWSALAPDFEEVEENVDYLEKEDEFDEVDEAEQQQ---GLEQEEEIAIDLRTRE
```

Supplementary Figure 3

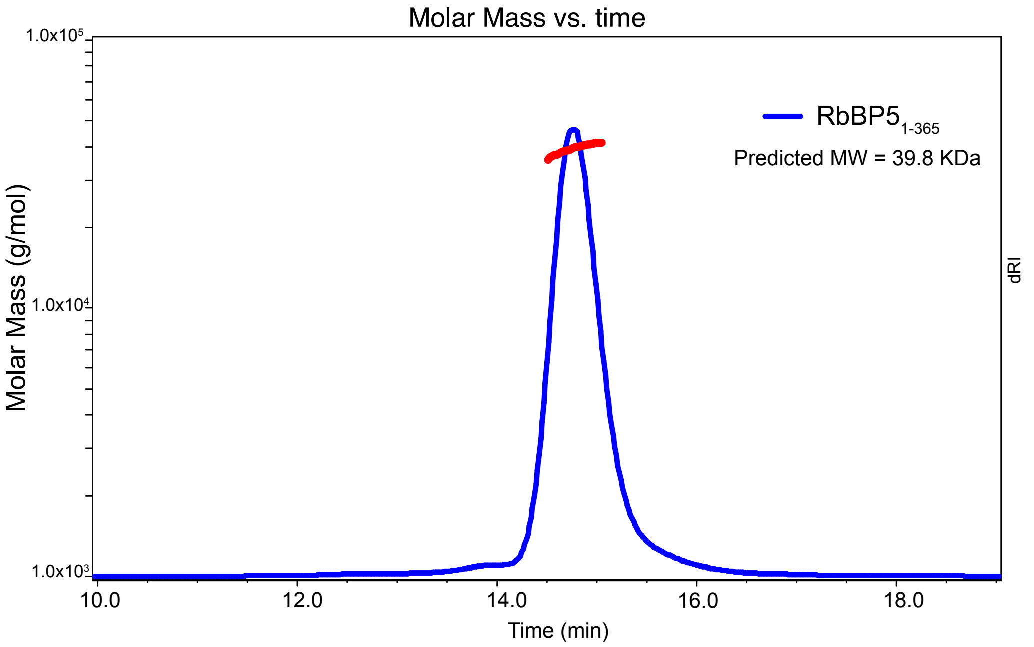

Molar mass moments (g/mol)

Mn = 3.940×104 (±0.796%)
Mw = 3.945×104 (±0.770%)
Mz = 3.951×104 (±1.700%)

Polydispersity

Mw/Mn = 1.001 (±1.107%)
Mz/Mn = 1.003 (±1.877%)
